# Finite Element Analysis of Residual Stress Distribution Patterns of Prestressed Composites Considering Interphases

**DOI:** 10.3390/ma16041345

**Published:** 2023-02-05

**Authors:** Meng Wang, Xiaochen Hang

**Affiliations:** 1 School of Mechanics & Civil Engineering, China University of Mining and Technology, Xuzhou 221116, China; 2 College of Mechanical and Electronic Engineering, Nanjing Forestry University, Nanjing 210037, China

**Keywords:** residual stress, finite element analysis, prestressed composites, CTE of interphase, elastic modulus of interphase

## Abstract

New finite element analysis procedures are developed in this study to obtain the precise stress distribution patterns of prestressed composites. Within the FEM procedures, an equivalent thermal method is modified to realize the prestress application, and a multi-step methodology is developed to consider coupling effects of polymer curing and prestress application. Thereafter, the effects of interphases’ properties, including the elastic modulus and coefficient of thermal expansion (CTE), on the stress distribution patterns are revealed. Analytical methods for residual stress prediction are modified in this study to demonstrate the finite element analysis procedures. From the residual stress results, it is found that the increase in the prestress level tends to contribute to the initiation of interphase debonding. The increase in the elastic modulus or CTE of the interphase results in very large circumferential and axial stress values appearing in the interphase. When the elastic modulus in the interphase is heterogeneous, the predicted stress values in the fiber and matrix are similar to the results predicted with the equivalent elastic modulus of the interphases. However, the heterogeneous elastic modulus results in serious circumferential and axial stress gradients in the interphase.

## 1. Introduction

Prestressed composites have been developed and widely used in civil engineering [1,2]. The properties of composites are believed to be dependent on the residual stress and internal structures, which are induced by the manufacturing process. The existence of residual stress is critical for the damage initiation, damage evolution, and strength values of composites [3,4,5,6]. Thus, it is necessary to reveal the changes of residual stress distribution patterns in prestressed composites. For the prestressed composites, the manufacturing process is composed of preload application, a polymer curing process, and a preload release process [6,7,8,9,10]. Thus, the residual stress of prestressed composite is composed of not only the thermal residual stress (TRS) induced by polymer curing, but also the residual stress induced by the prestress application process.

If the polymer is cured under high-temperature conditions, thermal residual stress is inevitable due to the mismatch of coefficients of thermal expansion (CTE). TRS is very important for the damage initiation and propagation analyses of the composites [3,4]. To obtain the TRS in the composites, both analytical and numerical methods have been developed and widely used. The developed analytical methods can be divided into two groups, including one-dimensional models [11,12] and three-dimensional models [13,14]. For the one-dimensional models, only the TRS values along the fiber direction can be obtained. For the three-dimensional models, the calculation is usually conducted under the cylindrical coordinates, and the TRS values at the radial, circumferential, and axial directions can be calculated. The geometrical models for the analytical methods need to be simplified before the calculation. Among the numerical methods, the FEM method is the most widely used [15,16,17]. With the periodical boundary conditions, the TRS distribution patterns of composites have been analyzed [18,19,20]. In [18,19,20], after completion of the TRS analysis, the obtained TRS values were set as the initial conditions for the damage analysis of the composites.

To evaluate residual stress in the prestressed composites with numerical methods, not only the thermal residual stress should be considered, but also the prestress application method should be developed. Many numerical methods for prestress application have been developed and used in civil engineering [21,22,23,24] and manufacturing engineering [25,26]. Within civil engineering, the thermal method was widely used for prestress application in steel. Within manufacturing engineering, the multi-step methodology was widely adopted to apply prestress. The thermal method was used for the residual stress prediction in prestressed composites without considering polymer curing [27]. Furthermore, an analytical method based on spring theory was developed to demonstrate the FEM analysis results in [27]. To predict the residual stress in prestressed composites considering both thermal loading and prestress application, analytical methods have been developed [11,12]. However, there are less numerical studies focusing on how to obtain the residual stress at different directions in prestressed composites considering both polymer curing and prestress application.

Among the models analyzed above, the interphase is usually ignored due to geometry simplification. The interphase is very important for the damage initiation and strength values of the composites [28,29]. Thus, it is necessary to evaluate the factors influencing stress values in the interphase. The effects of the CTE and elastic modulus of the interphase on the thermal residual stress distribution patterns of composites have been evaluated [15]. In [15], the CTE and elastic modulus values are assumed to be uniform in the interphase. However, the elastic modulus of the interphase is believed to be heterogeneous in [30,31,32,33,34]. Furthermore, the coupling effects of the interphase and prestress on the residual stress distribution in composites have not been evaluated.

In this study, to reveal the effects of prestress level and properties of interphase on the residual stress of unidirectional composites, new finite element analysis procedures are developed with both polymer curing and prestress application considered. In the first section, the finite element model to be analyzed is presented. Then, residual stress prediction methods are presented including a modified analytical method and numerical methods. Among the numerical methods, the equivalent thermal method is modified to apply the prestress, and the multi-step methodologies are developed to consider both polymer curing and prestress application. The FEM analysis procedures will be demonstrated with the analytical methods. Finally, the effects of prestress and the interphase’s properties on the stress distribution patterns are evaluated.

## 2. Finite Element Model

**Figure 1:**
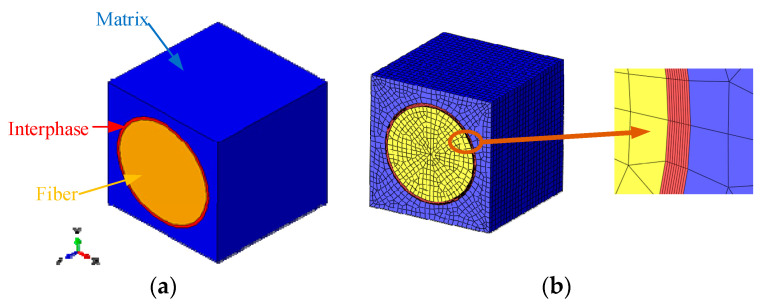
RVE model considering interphase for prestressed composites. (**a**) Geometrical model; (**b**) finite element model.

The representative volume element (RVE) model with fiber square distribution pattern (Figure 1) is adopted here to reveal the effects of prestress and the interphase’s properties on the residual stress distribution of prestressed composites. The glass fiber and polymer matrix’s elastic modulus and CTE parameters of the prestressed composites in [19] are adopted in this study to conduct the residual stress analyses. The constituents’ mechanical property parameters are shown in Table 1. The fiber volume fraction is set to be 40%, and the fiber diameter is set to be 15 μm.

To consider the interphases in the composites, the RVE model with interphases around the fibers is established. The thickness of the interphase is set to be 500 nm [33]. In [33], the material parameters of the interphase are set to be homogenized. The elastic modulus is set to be 1.9 GPa. This elastic modulus value is adopted in this study as a reference. The Poisson’s ratio of the interphase is assumed to be same as that of matrix. The approximate mesh size for the RVE is set to 0.4 in ABAQUS (Figure 1b). The mesh type for the fiber and matrix contains both C3D8R and C3D6 elements. For the interphase, only the C3D8R element is used.

**Figure 2:**
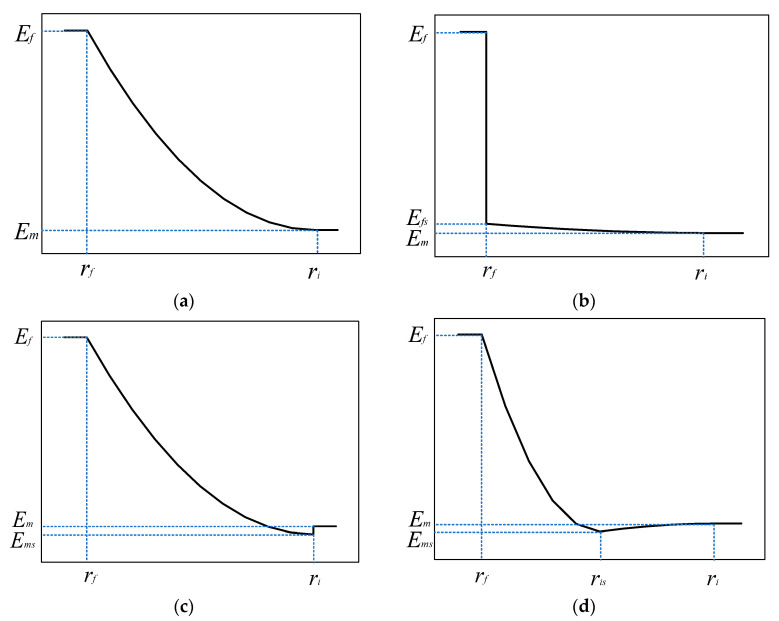
Illustrations for the distribution patterns of elastic modulus. (**a**) *α* = 1, *β* = 1, *γ* = 1; (**b**) *α* = 0.1, *β* = 1, *γ* = 1; (**c**) *α* = 1, *β* = 0.1, *γ* = 1; (**d**) *α* = 1, *β* = 0.1, *γ* = 0.5.

If the elastic modulus values in the interphase are heterogeneous, the typical elastic modulus distribution patterns from the fiber to the matrix can be expressed as exponential variation (Equations (1)–(3)) [34]. Four different elastic modulus distribution patterns within the interphase will be considered in this study. For case 1, shown in Figure 2a, the lowest elastic modulus’s location is at the interphase’s boundary, and the lowest elastic modulus value is the matrix’s elastic modulus. For case 2 (*α* = 0.1), the fiber interface bond strength is weak, and the elastic modulus at the fiber boundary is set to be a very small value, which corresponds to Figure 2b. For case 3 (*β* = 0.1), the matrix solidification is not good and fiber bonding is perfect. The corresponding elastic distribution patterns are shown in Figure 2c. For the last case (*β* = 0.1, *γ* = 0.5), the illustration is shown in Figure 2d. It can be observed that the elastic modulus firstly decreases from the fiber’s boundary to the lowest boundary and gradually increases to the interphase’s boundary.

(1) $$E_{i}=\{\begin{array}{ll} \beta E_{m}+\left(\alpha E_{f}-\beta E_{m}\right)R\left(r\right) & r_{f}<r<\gamma \left(r_{i}-r_{f}\right)+r_{f}\\ E_{m}+\left(\beta E_{m}-E_{m}\right)Q\left(r\right) & \gamma \left(r_{i}-r_{f}\right)+r_{f}<r<r_{i} \end{array}$$

where $\alpha =E_{fi}/E_{f}$, $\beta =E_{ms}/E_{m}$, $\gamma =\left(r_{is}-r_{f}\right)/\left(r_{i}-r_{f}\right)$. Thus, $r_{is}=\gamma \left(r_{i}-r_{f}\right)+r_{f}$.

(2) $$R\left(r\right)=\frac{1-\left(r/r_{is}\right)\exp(1-r/r_{is})}{1-\left(r_{f}/r_{is}\right)\exp(1-r_{f}/r_{is})}$$

(3) $$Q\left(r\right)=\frac{1-\left(r/r_{i}\right)\exp(1-r/r_{i})}{1-\left(r_{is}/r_{i}\right)\exp(1-r_{is}/r_{i})}$$

**Figure 3:**
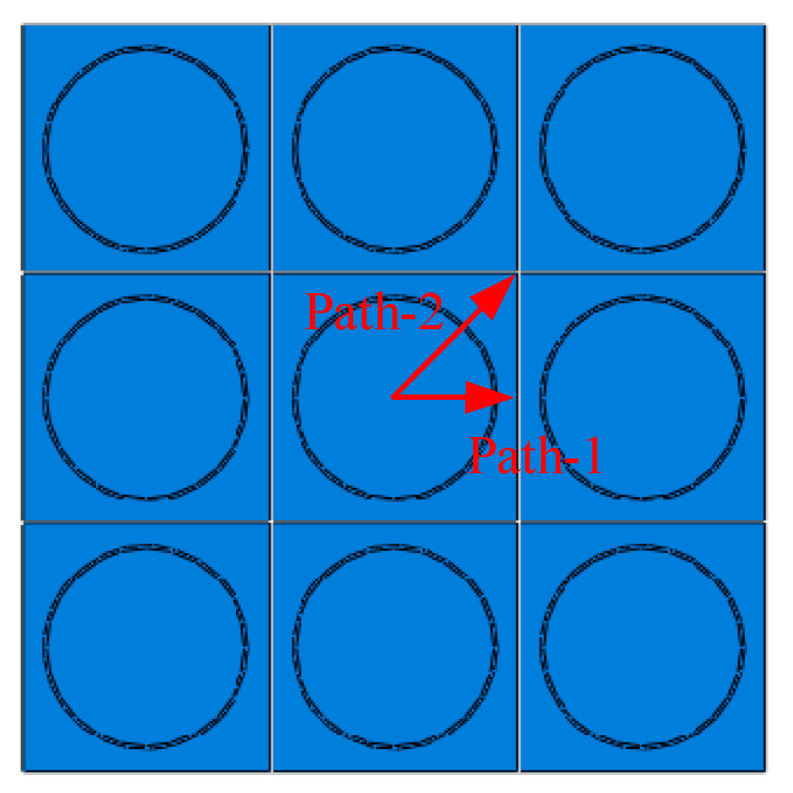
Illustration for the inter-fiber directions.

The periodical boundary conditions (PBCs) are adopted in this study to ensure the continuity of the traction and displacement between adjacent RVEs. More details about the PBCs can be found in [35]. From the fiber distribution patterns, two typical inter-fiber directions can be found, including path-1 and path-2 (Figure 3).

## 3. Residual Stress Prediction Methods

### 3.1. Theoretical Method

The analytical method based on the spring model [27] is adopted to demonstrate the capability of the FEM method to predict residual stress induced by applying prestress. With the model, the residual stress values in the fiber and matrix can be obtained with Equations (4) and (5). More details about the spring model can be found in [27].

(4) $$\sigma_{f}^{res}=\sigma_{pre}-\frac{\sigma_{pre}}{1+\left(\frac{E_{m}}{E_{f}}\right)\left(\frac{1-V_{f}}{V_{f}}\right)}$$

(5) $$\sigma_{m}^{res}=-\frac{\sigma_{pre}}{\left(\frac{E_{m}}{E_{f}}\right)+\left(\frac{1-V_{f}}{V_{f}}\right)}$$

where *σ_pre_* is the prestress value, $\sigma_{f}^{res}$ is the residual stress in the fiber, $\sigma_{m}^{res}$ is the residual stress in the matrix along the longitudinal direction, *V_f_* is the fiber volume fraction, *E_m_* is the elastic modulus of the matrix, and *E_f_* is the elastic modulus of the fiber.

**Figure 4:**
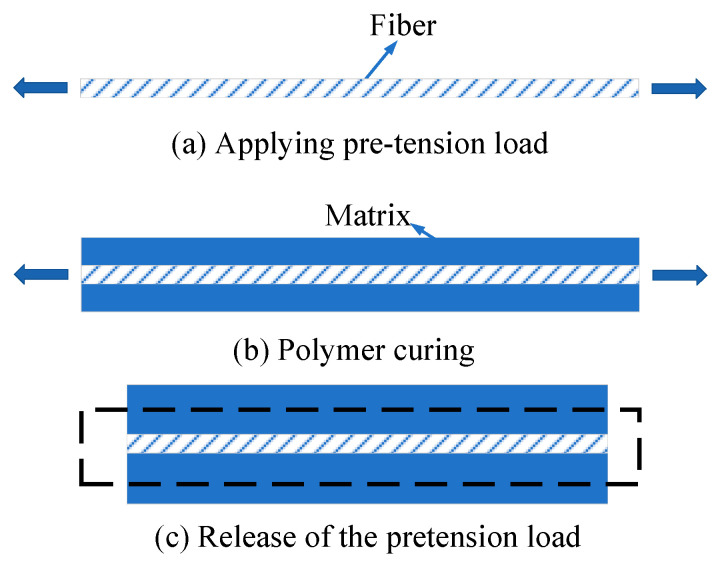
Manufacturing process of prestressed composites.

As the prestressed composites are manufactured considering both polymer curing and prestress application (Figure 4), the effects of the TRS on the residual stress values should be considered. The analytical method for the residual stress prediction of prestressed composites was developed in [11], considering both thermal loading and prestress application. It is adopted in this study to demonstrate the effectiveness of the FEM analysis procedures to be developed in this study. The equations for predicting residual stress values of prestressed composites along the longitudinal direction are shown in Equations (6)–(8).

(6) $$\sigma_{f}=E_{f}\left(\varepsilon_{1}^{p}-\alpha_{f}\Delta T\right)+\sigma_{f}^{p}$$

(7) $$\sigma_{m}=E_{m}\left(\varepsilon_{1}^{p}-\alpha_{m}\Delta T\right)$$

(8) $$\varepsilon_{1}^{p}=\frac{-\sigma_{f}^{p}V_{f}}{E_{11}}+\alpha_{11}\Delta T$$

where, $\sigma_{f}^{p}$ is the prestress value applied in the fiber, $\varepsilon_{1}^{p}$ is the preload strain, *E*_11_ and *α*_11_ are the equivalent elastic modulus and thermal expansion coefficient of the composite along the longitudinal direction, respectively. *E*_11_ and *α*_11_ can be calculated with Equations (9) and (10), respectively.

(9) $$E_{11}=V_{f}E_{f}+V_{m}E_{m}$$

(10) $$\alpha_{11}=\frac{\alpha_{f}V_{f}E_{f}+\alpha_{m}V_{m}E_{m}}{V_{f}E_{f}+V_{m}E_{m}}$$

Based on the same methodology, it can be deduced that the analytical model for the residual stress prediction considering the interphase can be obtained with Equation (11). Furthermore, the new preload strain should be recalculated due to the change of the equivalent elastic modulus and thermal expansion coefficient of the composite along the longitudinal direction following Equations (12) and (13).

(11) $$\sigma_{i}=E_{i}\left(\varepsilon_{1}^{p}-\alpha_{i}\Delta T\right)$$

(12) $$E_{11}=V_{f}E_{f}+V_{m}E_{m}+V_{i}E_{i}$$

(13) $$\alpha_{11}=\frac{\alpha_{f}V_{f}E_{f}+\alpha_{m}V_{m}E_{m}+\alpha_{i}V_{i}E_{i}}{V_{f}E_{f}+V_{m}E_{m}+V_{i}E_{i}}$$

If the heterogenous interphase’s modulus patterns are considered, the analytical method can be modified further. The interphase regions are assumed to be divided into *J* layers and the elastic modulus and CTE within each layer are set to be the same. With this assumption, the stress values within each interphase layer *j* can be calculated with Equation (14). The equivalent elastic modulus and CTE of composites should also be updated with Equations (15) and (16), respectively, to calculate the preload strain.

(14) $$\sigma_{i}^{j}=E_{i}^{j}\left(\varepsilon_{1}^{p}-\alpha_{i}^{j}\Delta T\right)\;j=1,2,\ldots ,J$$

(15) $$E_{11}=V_{f}E_{f}+V_{m}E_{m}+V_{i}^{j}E_{i}^{j}\;j=1,2,\ldots ,J$$

(16) $$\alpha_{11}=\frac{\alpha_{f}V_{f}E_{f}+\alpha_{m}V_{m}E_{m}+\alpha_{i}^{j}V_{i}^{j}E_{i}^{j}}{V_{f}E_{f}+V_{m}E_{m}+V_{i}^{j}E_{i}^{j}}\;j=1,2,\ldots ,J$$

### 3.2. Numerical Method

Without considering polymer curing, two different numerical methods will be compared in this study to apply the fiber prestress. For the first method, the multi-step methodology is adopted, which means that the calculation process obeys the prestress application process shown in Figure 4. Firstly, the pretension load is applied at the fiber without considering the matrix. Then, the stress distribution patterns in the fiber are set as the initial state for the RVE model considering both fiber and matrix. Finally, the stress distribution patterns after the release of pretension loading can be obtained with the static analysis.

The second method is modified from the equivalent thermal method. In the original equivalent thermal method widely used in prestressed-steel-reinforced concrete, the prestress is applied directly using the CTE of steel through applying an equivalent temperature loading to the steel. The transverse expansion of steel induced by the pretension is omitted due to the very small volume fraction. However, for the composites, the volume fraction of reinforcement is comparable with the matrix’s volume fraction generally. Thus, the transverse expansion of fibers induced by the release of pretension loading should be considered for prestressed composites.

To modify the equivalent thermal method, firstly, the CTE of fibers along the longitudinal direction is set as the same as that used for the thermal residual stress analysis. Secondly, the equivalent temperature loading is calculated as Δ*T* = *σ_pre_*/(*αE_f_*). Thirdly, the transverse CTE of the fibers should be defined as *α_ft_* = *ε_ft_*/Δ*T*. *ε_ft_* represents the transverse expansion strain of the fiber under the equivalent temperature loading and it can be obtained based on the results predicted with the multi-step simulation method. Finally, once the CTE along the transverse direction is calculated, it can be used at the other prestress level.

**Figure 5:**
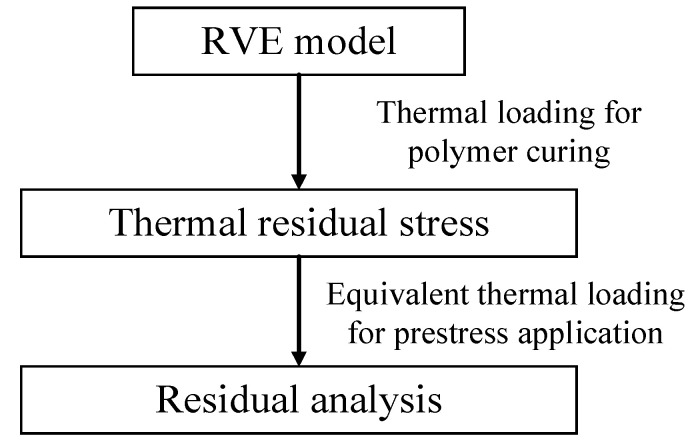
Multi-step methodology for the prediction of residual stress.

To obtain the residual stress distribution patterns of prestressed composites with FEM considering both polymer curing and prestress application, a multi-step methodology based on the manufacturing processes is developed here (Figure 5). Firstly, the TRS analysis considering the polymer curing is conducted based on the difference between curing temperature and room temperature. Then, the thermal residual stress values are set as the initial stress states for the RVE model. Thereafter, the transverse CTE of the fiber is set in the RVE and the equivalent temperature loading is calculated based on the prestress value, longitudinal CTE, and elastic modulus of the fiber. Finally, the residual stress states of the prestressed composites can be obtained. All the numerical analyses are conducted with the commercial software Abaqus/Standard 6.14. Due to the consideration of the transverse expansion of fibers based on the multi-step methodology, the developed numerical method should be available for the other kinds of fiber-reinforced composites.

## 4. Results and Discussion

### 4.1. Model Verification

The thermal loading and prestress level from [8] are adopted in this study to analyze the residual stress distribution patterns in prestressed composites. The temperature drop for the thermal loading equals 2.4 °C and the applied prestress is 60 MPa.

**Figure 6:**
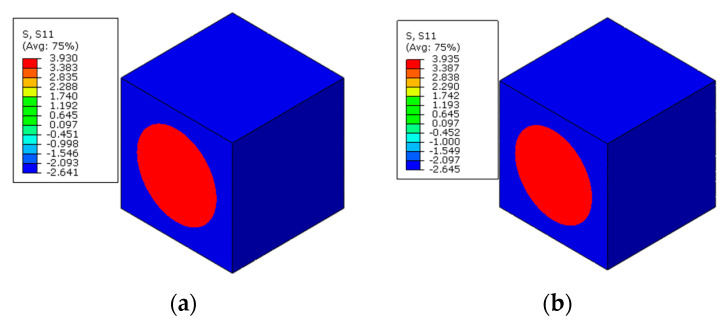
Residual axial stress induced by the prestress application (**a**) with the multi-step method and (**b**) with the modified equivalent thermal method.

**Figure 7:**
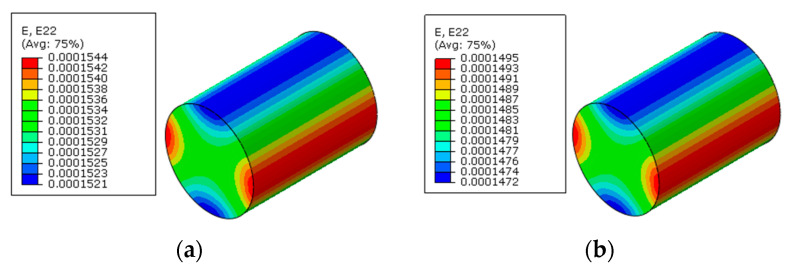
Transverse strain values in the fiber after prestress application (**a**) with the multi-step method and (**b**) with the modified equivalent thermal method.

The numerical methods for prestress application are first verified here, which means that polymer curing is not considered. With the first prestress application method, the longitudinal stress distribution patterns of the RVE after applying prestress are shown in Figure 6a. It can be found that after the release of pretension load, the matrix is in a compression state and the fiber is still in a tension state along the fiber direction. From the transverse strain distribution patterns of the fiber (Figure 7a), it can be found that the fiber expands along the transverse direction.

**Figure 8:**
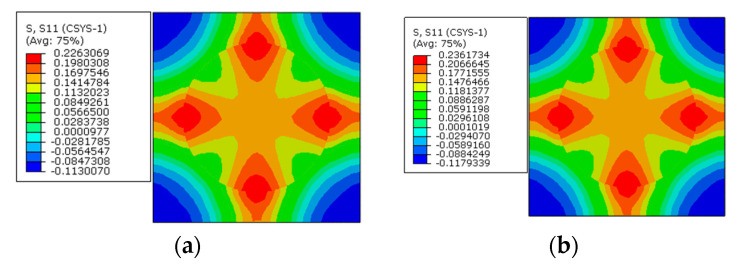
Radial stress distribution after prestress application (**a**) with the multi-step method and (**b**) with the modified equivalent thermal method.

**Figure 9:**
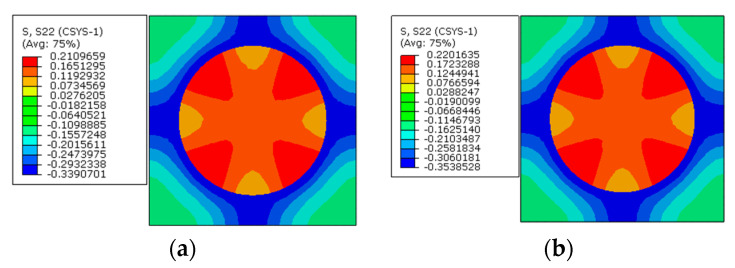
Circumferential stress distribution after prestress application (**a**) with the multi-step method and (**b**) with the modified equivalent thermal method.

To apply the designed prestress with the second prestress application method, the equivalent temperature loading applied to the fibers is first calculated as 165.5 °C, based on the longitudinal CTE of fiber. Then, based on the transverse strain values of the fiber predicted with the first method, the equivalent transverse CTE of the fiber for prestress application is calculated as −9.5 × 10^−7^/°C. Finally, with the longitudinal and equivalent transverse CTEs of the fiber, the stress distribution patterns calculated with the modified equivalent thermal method are shown in Figure 6b. The stress values in the fiber and matrix are almost the same as those obtained with the first method. However, there is a little difference between the predicted transverse strain values of the fibers (Figure 7a,b), which should be attributed to the calculation error induced during the calculation of the equivalent transverse CTE of the fiber. From the analytical method developed in [27], the axial stress values in the matrix and fiber are calculated as −2.5 MPa and 3.8 MPa, respectively. The values are very close with those predicted with FEM (Table 2), which demonstrates the effectiveness of the prestress application methods. Furthermore, the radial and circumferential stress distribution patterns in the RVE are shown in Figure 8 and Figure 9 with different prestress application methods. The consistency between the stress distribution patterns also demonstrates the effectiveness of the prestress application method. As the modified equivalent thermal method is more convenient than the first method, the equivalent thermal method will be adopted for the analyses blow.

**Figure 10:**
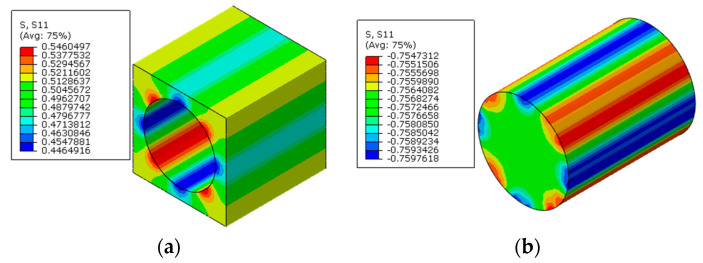
Thermal residual stress distribution patterns of composites (**a**) in the matrix and (**b**) in the fiber.

The multi-step FEM methodology for predicting residual stress values of prestressed composites considering both polymer curing and prestress application is demonstrated here. Following the multi-step analysis procedures, the thermal residual analysis is conducted first. The thermal residual stress distribution patterns along the longitudinal direction are shown in Figure 10. It can be observed that within the fibers, the stress values along the longitudinal direction are almost uniform. However, in the matrix, the stress values between two fibers with different inter-fiber distances are non-uniform. The fiber is in a compressive state and the matrix is in a tensile state along the fiber direction.

**Figure 11:**
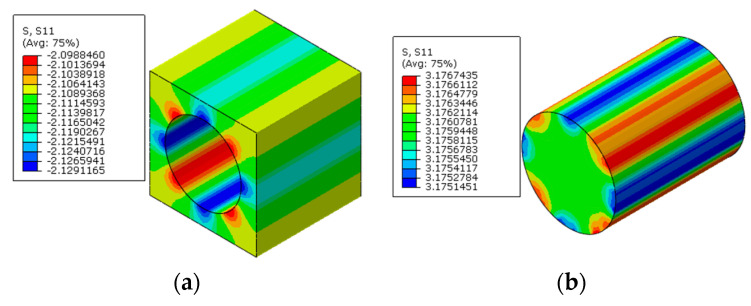
Residual stress distribution patterns of prestressed composites (**a**) in the matrix and (**b**) in the fiber.

Based on the thermal residual stress distribution patterns, with the modified equivalent thermal method, the residual stress distribution patterns induced by both polymer curing and prestress application along the longitudinal direction are shown in Figure 11. It can be observed that the stress distribution pattern remains unchanged within each constituent under the applied prestress. The fiber is in a tensile state and the matrix is in a compressive state, which is the same as the conclusion in [8]. The average stress value in the matrix along the axial direction is −2.11 MPa and the average value in the fiber is 3.17 MPa (Table 3). The values are similar with those predicted with the analytical method [11] (−2.14 MPa for the matrix and 3.2 MPa for the fiber), which demonstrates the effectiveness of the multi-step analysis procedures for the residual stress prediction of prestressed composites.

### 4.2. Model Considering Interphase

**Figure 12:**
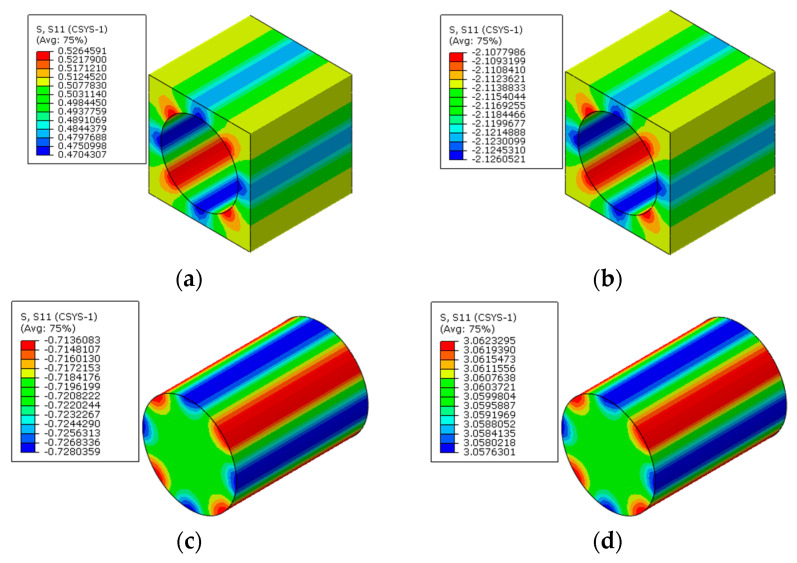

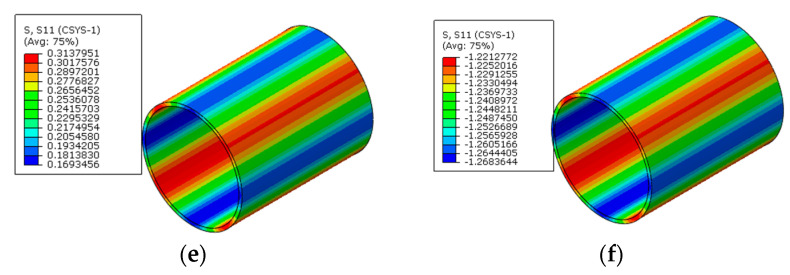
Residual axial stress distribution patterns in the RVE considering interphase (**a**) in the matrix before applying prestress; (**b**) in the matrix after applying prestress; (**c**) in the fiber before applying prestress; (**d**) in the fiber after applying prestress; (**e**) in the interphase before applying prestress; (**f**) in the interphase after applying prestress.

To reveal the effects of interphases on the residual stress of prestressed composites, the models with the interphase are analyzed. In this section, the elastic modulus of the interphase is set to be 1.9 GPa and the CTE of the interphase is set to be the CTE of the matrix. The temperature drop for the thermal loading is also set to be 2.4 °C and the applied prestress is 60 MPa. The axial stress distribution patterns of the model considering the interphase are shown in Figure 12.

Compared with the models that do not consider the interphase, the axial stress distribution patterns in the RVE are almost the same. The interphase is in a tensile state when only the polymer curing is considered. After considering the prestress, the interphase is in a compressive state, which is similar to the matrix. Based on the modified analytical method considering the interphase, the equivalent longitudinal stress values in the matrix, fiber, and interphase are obtained as −2.14 MPa, 3.03 MPa, and −1.22 MPa, respectively. The values are very close with the results predicted with the FEM method (Table 4), which demonstrates the effectiveness of the numerical method once again.

**Figure 13:**
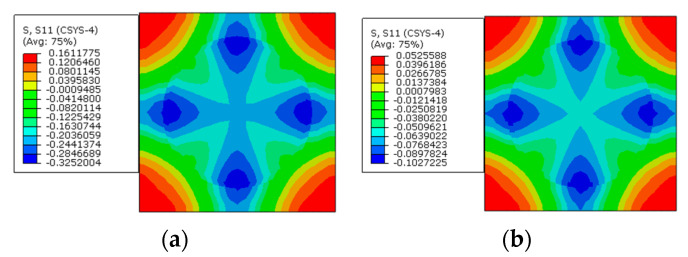
Stress distribution patterns along the radial direction (**a**) before applying prestress and (**b**) after applying prestress.

The stress distribution patterns along the radial direction are shown in Figure 13. The matrix region between two fibers with smaller inter-fiber distance is in a compressive state and the region between two fibers with larger inter-fiber distance is in a tensile state. It can be observed that the applied prestress does not change the stress distribution patterns.

**Figure 14:**
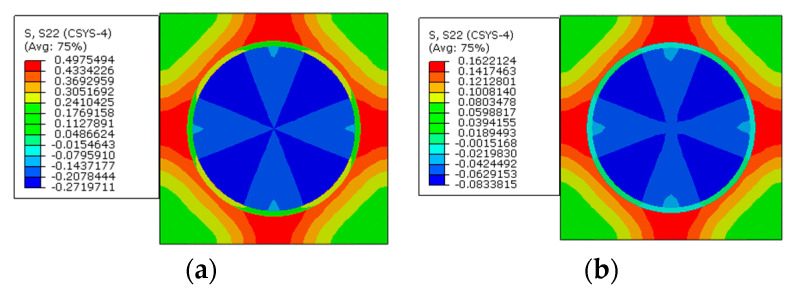
Stress distribution patterns along the circumferential direction (**a**) before applying prestress and (**b**) after applying prestress.

The stress distribution patterns along the circumferential direction are shown in Figure 14. The stress in the matrix concentrates between the fibers with less inter-fiber distance, and the matrix is in a tensile state. The fiber is in a compressive state. The application of the designed prestress also does not change the stress distribution patterns seriously.

### 4.3. Effects of Prestress Level

**Figure 15:**
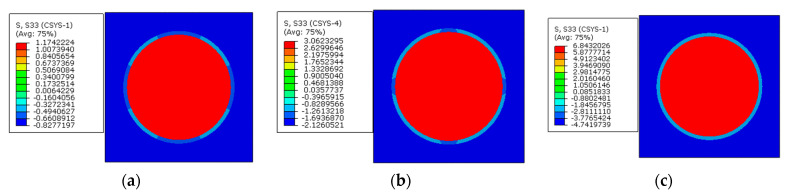
Axial stress distribution patterns with different prestress values. (**a**) 30 MPa; (**b**) 60 MPa; (**c**) 120 MPa.

**Figure 16:**
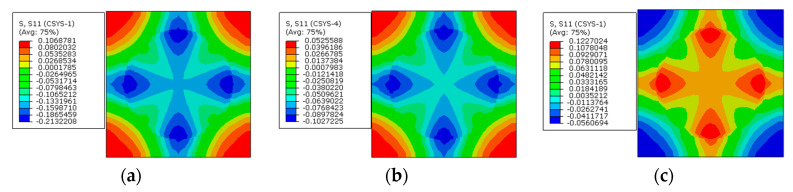
Radial stress distribution patterns with different prestress values. (**a**) 30 MPa; (**b**) 60 MPa; (**c**) 120 MPa.

**Figure 17:**
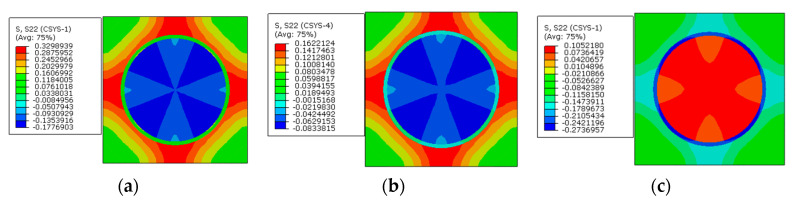
Circumferential stress distribution patterns with different prestress values. (**a**) 30 MPa; (**b**) 60 MPa; (**c**) 120 MPa.

Based on the model considering the interphase analyzed above, different prestress values (30 MPa, 60 MPa, and 120 MPa) are considered here. The stress distribution patterns of the RVE are shown in Figure 15, Figure 16 and Figure 17. Along the axial direction, the stress distribution patterns are very similar with each other. The axial tensile stress values in the fiber and compressive stress values in the matrix and interphase increase with the increase in the prestress value. With the increase in the differences between axial stress values in the fiber and matrix, it can be deduced that longitudinal shear stress in the interphase would increase based on the method developed in [36].

For the radial stress distribution patterns (Figure 16), the increase in the prestress changes the stress concentration regions. The tensile radial stress concentration region changes from the matrix to the fiber and the compressive stress concentration region changes from the fiber to the matrix. Compared with the thermal residual stress distribution pattern (Figure 13a), it can be found that a small prestress value cannot change the radial stress distribution induced by the polymer curing. Compared with the stress distribution patterns with only prestress applied (Figure 8), it can be deduced that a large prestress value will dominate the stress distribution pattern, which is attributed to the large transverse expansion value of the fiber due to the release of pretension loading. Especially for the interphase where the damage initiates under external loading, the radial stress state changes from the compressive state to the tensile state. Combined with the increase in the longitudinal shear stress, it can be deduced that a large prestress level would result in the interphase debonding according to the widely used failure criteria of the interphase [37]. The debonding of the interphase is believed to be responsible for the decrease in the strength values after applying a large prestress value [38].

Along the circumferential direction (Figure 17), the stress distribution patterns are also changed by the prestress level. The changes of tensile stress concentration region are similar to the changes along the radial direction. However, the compressive stress concentration region changes from the matrix to the interphase. With a small prestress level, the circumferential stress distribution pattern is similar to the distribution patterns with only polymer curing considered (Figure 9). If a large prestress level is applied, the stress distribution patterns are governed by the prestress, which illustrates the importance of prestress level on the circumferential stress distribution patterns. However, for the circumferential stress values in the interphase, the change of the stress state is opposite to the radial stress, which means that the stress changes from the tensile state to the compressive state. It is believed that there is a certain prestress level that will result in a near-zero circumferential stress state in the interphase.

### 4.4. Effects of Interphase’s CTE

To reveal the effects of the interphase’s CTE on the residual stress values of prestressed composites, based on the model considering the interphase analyzed above, three different CTE values (*α_i_* = 0.1*α_m_*, 1.0*α_m_*, 10*α_m_*) are considered. The elastic modulus of the interphase is set to be 1.9 GPa. The prestress level (60 MPa) is believed to be the beneficial value under the given polymer curing condition [8], so the prestress level is adopted for the analyses below. The residual stress distribution patterns of the models without and with considering prestress are all shown below along different directions. To reveal the effects of the interphase’s CTE on the stress values in the RVE more clearly, the stress values along two different fiber inter-fiber paths are also extracted and shown below.

**Figure 18:**
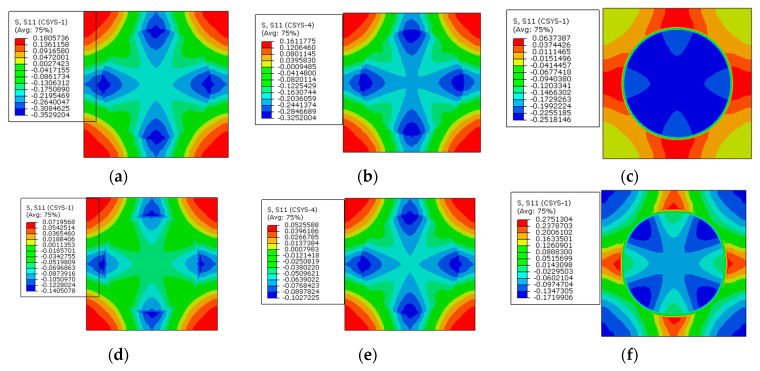
Radial stress distribution patterns. (**a**) *α_i_* = 0.1*α_m_* without prestress; (**b**) *α_i_* = 1.0*α_m_* without prestress; (**c**) *α_i_* = 10*α_m_* without prestress; (**d**) *α_i_* = 0.1*α_m_* with prestress; (**e**) *α_i_* = 1.0*α_m_* with prestress; (**f**) *α_i_* = 10*α_m_* with prestress.

The radial stress distribution patterns of the models without and with applying prestress are shown in Figure 18. It can be observed that the tensile and compressive stress concentration regions are changed due to the increase in the interphase’s CTE. However, compared with the effects of prestress level, the stress concentration region still remains within the corresponding constituent. With the application of prestress, it can be observed that except the condition *α_i_* = *α_m_*, the stress distribution patterns are changed by the prestress applied.

**Figure 19:**
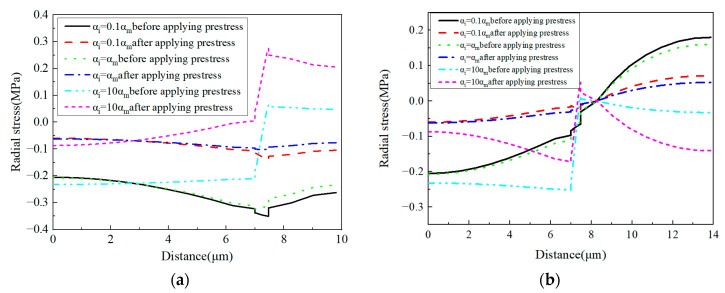
Radial stress values with different CTEs (**a**) along path-1 and (**b**) along path-2.

For the radial stress values (Figure 19) along path-1, with the increase in the CTE, the stress gradient in the interphase changes from the negative state to the positive state. The stress in the matrix changes from the compressive state to the tensile state. However, along path-2, the stress gradient within the interphase remains positive and the stress gradient value increases with the increase in the CTE. Furthermore, the stress in the matrix changes from the tensile state to the compressive state. The stress distribution in the fiber also redistributes due to the increase in the CTE.

**Figure 20:**
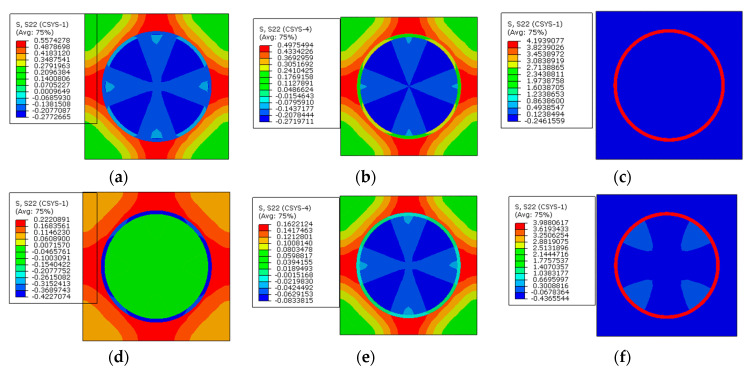
Circumferential stress distribution patterns. (**a**) *α_i_* = 0.1*α_m_* without prestress; (**b**) *α_i_* = 1.0*α_m_* without prestress; (**c**) *α_i_* = 10*α_m_* without prestress; (**d**) *α_i_* = 0.1*α_m_* with prestress; (**e**) *α_i_* = 1.0*α_m_* with prestress; (**f**) *α_i_* = 10*α_m_* with prestress.

Along the circumferential direction (Figure 20), with the increase in the CTE of the interphase, the tensile stress concentration region changes from the matrix to the interphase. The application of designed prestress reduces the tensile stress concentration value in the RVE. Similar to the radial stress distribution patterns, it can be observed that except the condition *α_i_* = *α_m_*, the stress distribution patterns are changed by applying prestress.

**Figure 21:**
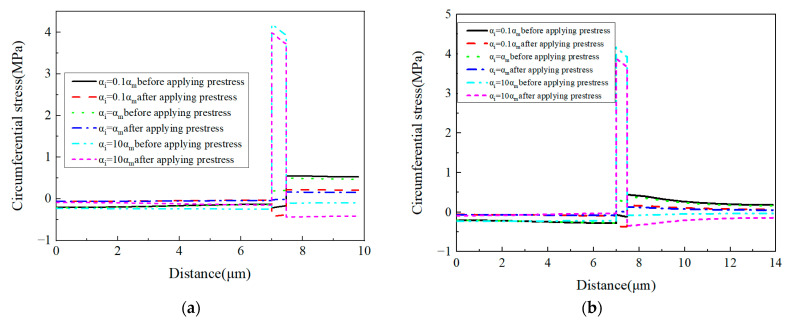
Circumferential stress values with different CTEs (**a**) along path-1 and (**b**) along path-2.

For the circumferential stress values (Figure 21), it can be found that the prestress and CTE of the interphase have very large effects on the circumferential stress values of the interphase. A large CTE value of the interphase results in a very large circumferential stress value in the interphase. Compared with the change of the interphase’s stress values, the change of the fiber’s stress values can be ignored. For the matrix, the increase in the CTE contributes to the decrease in the tensile circumferential stress. With a very large interphase CTE, the stress in the matrix is changed to the compressive state. The stress values along path-2 are similar to those along path-1, except that there is a small circumferential stress gradient within the matrix.

**Figure 22:**
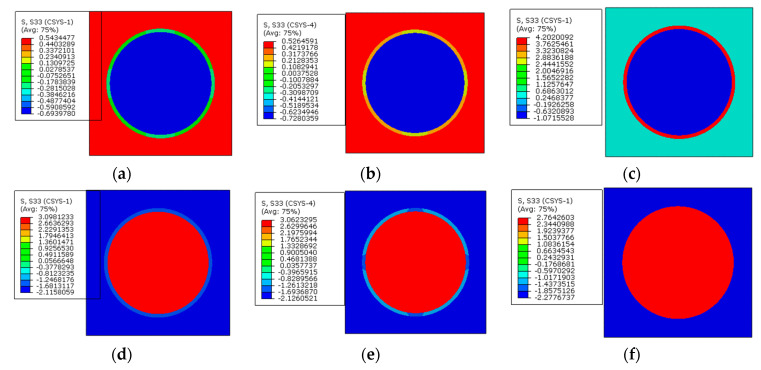
Axial stress distribution patterns. (**a**) *α_i_* = 0.1*α_m_* without prestress; (**b**) *α_i_* = 1.0*α_m_* without prestress; (**c**) *α_i_* = 10*α_m_* without prestress; (**d**) *α_i_* = 0.1*α_m_* with prestress; (**e**) *α_i_* = 1.0*α_m_* with prestress; (**f**) *α_i_* = 10*α_m_* with prestress.

Along the axial direction (Figure 22), it can be observed that with the increase in the CTE, the stress in the interphase is changed from the compressive state to the tensile state and the tensile stress values in the interphase are very large with a large interphase CTE. The stress values in the fiber and matrix are also changed, which should be attributed to the change of the interphase’s stress values.

**Figure 23:**
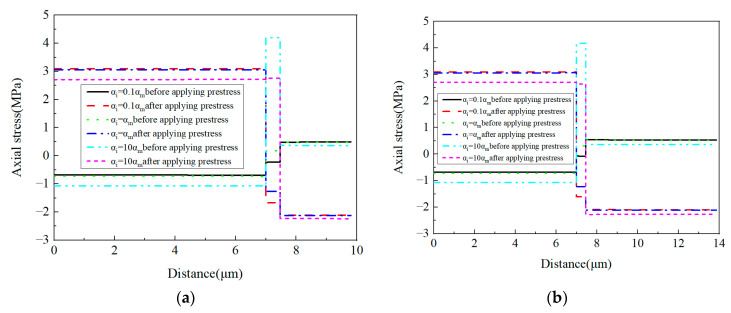
Axial stress values with different CTEs (**a**) along path-1 and (**b**) along path-2.

For the axial stress values (Figure 23), It can be observed that the increase in the CTE has little influence on the stress values in the matrix and has large effects on the fiber and interphase. Especially for the interphase, with the increase in the CTE, the stress values increase and the stress states change from the compressive state to the tensile state. Based on the balance requirement of the RVE, it can be deduced that due to the increase in the tensile stress in the interphase, both the compressive stress values in the fiber increase and the tensile stress values in the matrix decrease before applying prestress. After applying the given prestress, the increase in the CTE tend to change the stress state of the interphase from the tensile to the compressive state. The values along path-1 and path-2 are similar to each other.

Especially for the interphase, the max and min stress values in the interphase are shown in Table 5. It should be noted that the compressive radial stress would not result in the interface debonding at the microscale [37], so only the max radial stress is listed in Table 5. It can be observed that with the increase in the interphase’s CTE, the increase in the circumferential and axial stress in the interphase is much larger than that of the radial stress. Thus, it can be concluded that the circumferential and axial stress values in the interphase are more sensitive to the interphase’s CTE. The radial, circumferential, and axial stress in the interphase tend to change from the compressive to the tensile state with the increase in the interphase’s CTE before applying prestress. Furthermore, the circumferential and axial stress values are very similar to each other. However, after applying the prestress, the reduction in the axial stress is larger than the reduction in the circumferential stress in the interphase.

### 4.5. Effects of Interphase Elastic Modulus

To reveal the effects of the interphase elastic modulus on the residual stress of prestressed composites, based on the model considering the interphase analyzed above, three different interphase elastic modulus values are considered (*E_i_* = 1.9 GPa, 5 GPa, and 50 GPa). The stress distribution patterns predicted from the models are shown below at the cylindrical coordinate. Both the models with and without considering prestress are presented.

**Figure 24:**
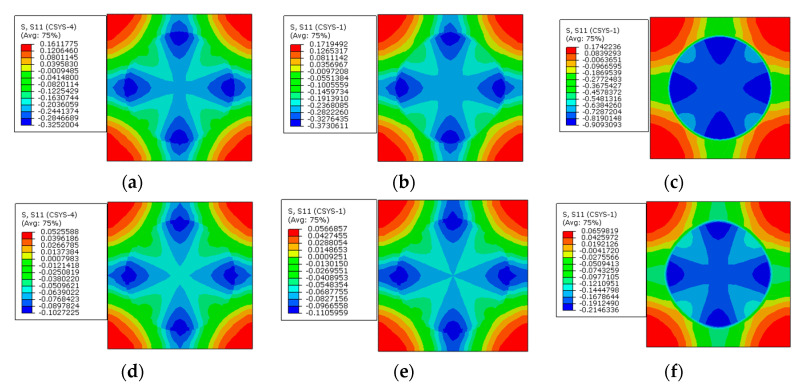
Radial stress distribution patterns. (**a**) *E_i_* = 1.9 GPa without prestress; (**b**) *E_i_* = 5 GPa without prestress; (**c**) *E_i_* = 50 GPa without prestress; (**d**) *E_i_* = 1.9 GPa with prestress; (**e**) *E_i_* = 5 GPa with prestress; (**f**) *E_i_* = 50 GPa with prestress.

The radial stress distribution patterns of the models without and with applying prestress are shown in Figure 24. It can be observed that the increase in the elastic modulus contributes to the change of the stress distribution patterns. The max tensile stress values in the matrix are very similar to each other. However, the max compressive stress value in the fiber increases. The application of the designed prestress has little influence on the stress distribution patterns, but the stress concentration values in the RVE are reduced.

**Figure 25:**
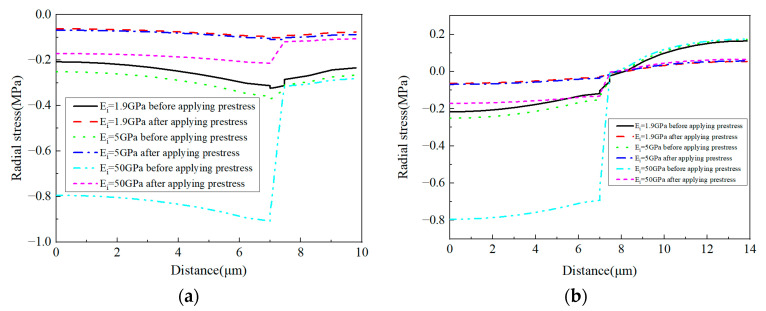
Radial stress values with different elastic modulus values (**a**) along path-1 and (**b**) along path-2.

For the radial stress values along path-1 (Figure 25a), it can be observed that all the radial stress values in the fiber, interphase, and matrix are in a compressive state. The increase in the interphase’s elastic modulus contributes to the decrease in the compressive stress in the RVE, especially for the fiber and interphase. Along path-2, most matrix regions are in a tensile state, which is different from the values along path-1. Compared with the fiber and interphase, the effects of the interphase’s elastic modulus on the radial stress of the matrix are much less. From Figure 25, it can also be observed that the stress concentration values in the RVE are decreased by the prestress.

**Figure 26:**
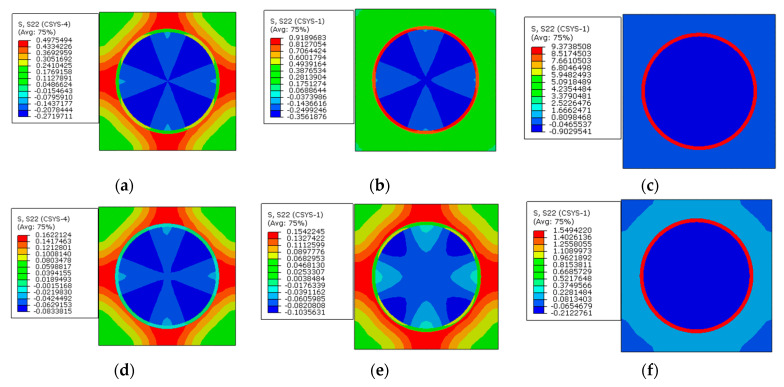
Circumferential stress distribution patterns. (**a**) *E_i_* = 1.9 GPa without prestress; (**b**) *E_i_* = 5 GPa without prestress; (**c**) *E_i_* = 50 GPa without prestress; (**d**) *E_i_* = 1.9 GPa with prestress; (**e**) *E_i_* = 5 GPa with prestress; (**f**) *E_i_* = 50 GPa with prestress.

From the circumferential stress distribution patterns (Figure 26), it can be observed that with the increase in the interphase’s elastic modulus, the tensile stress value in the interphase increases, which contributes to the change of the stress distribution patterns. The stress value in the interphase is decreased by the application of prestress. Furthermore, the max compressive stress value within one RVE is also reduced by the application of prestress.

**Figure 27:**
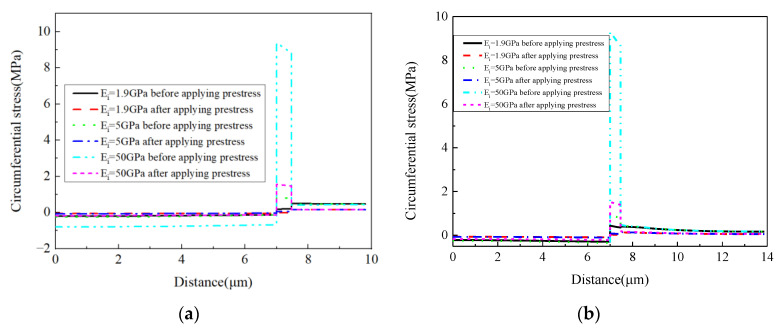
Circumferential stress values with different elastic modulus values (**a**) along path-1 and (**b**) along path-2.

The circumferential stress values along path-1 and path-2 are shown in Figure 27. With the increase in the elastic modulus of the interphase, the tensile circumferential stress in the interphase become larger under thermal loading. After applying the designed prestress, the differences between the stress values of the fiber and interphase become less. The reduction degree is much larger than the reduction induced by applying prestress on the models with different interphase CTE. It can also be observed that the elastic modulus of the interphase has little influence on the circumferential stress of the matrix. Compared with the values along path-1, the stress decreases along path-2 in the matrix regions, which is similar to the pattern shown in Figure 21.

**Figure 28:**
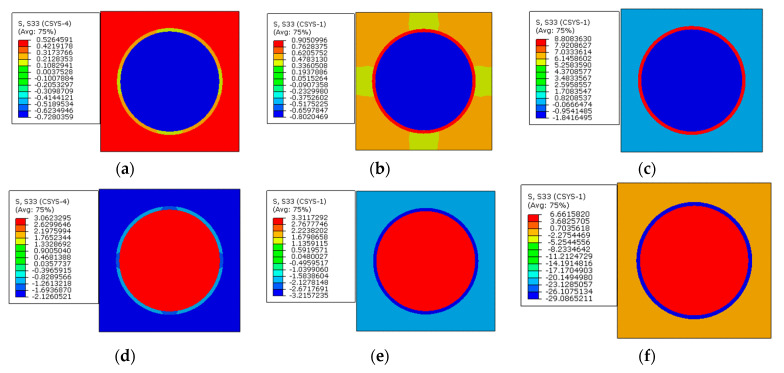
Axial stress distribution patterns. (**a**) *E_i_* =1.9 GPa without prestress; (**b**) *E_i_* =5 GPa without prestress; (**c**) *E_i_* =50 GPa without prestress; (**d**) *E_i_* = 1.9 GPa with prestress; (**e**) *E_i_* = 5 GPa with prestress; (**f**) *E_i_* = 50 GPa with prestress.

From the axial stress distribution patterns (Figure 28), it can be observed that the stress in one constituent is also almost uniform. With the increase in the interphase’s elastic modulus, the tensile stress value in the interphase increases with only thermal loading applied. After considering prestress, the stress in the interphase changes from the tensile state to the compressive state and the stress values also increase with the increase in the elastic modulus. The fiber is put in a tensile state after applying prestress and the tensile stress value becomes larger and larger with the increase in the interphase elastic modulus.

**Figure 29:**
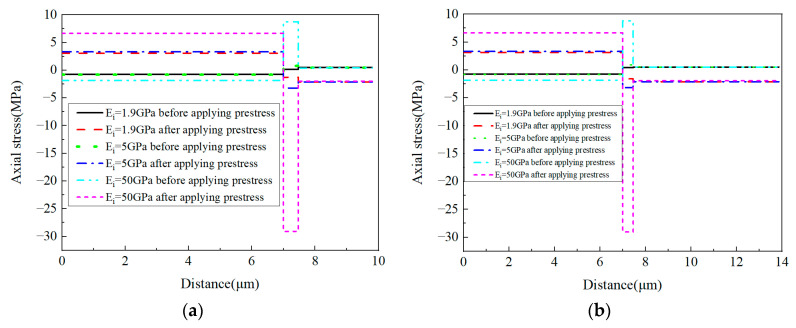
Axial stress values with different elastic modulus values (**a**) along path-1 and (**b**) along path-2.

With different interphase elastic modulus values, the axial stress values along path-1 and path-2 are shown in Figure 29. It can be observed that the elastic modulus of the interphase also has little influence on the matrix axial stress values. However, it has huge influences on the stress values of the fiber and the interphase. This is similar to the conclusion obtained in [15], which should be attributed to the small volume fraction of the interphase and smaller elastic modulus of the matrix compared with that of the fiber. With the increase in the elastic modulus, both the compressive stress in the fiber and the tensile stress in the interphase increase. The axial stress values along path-1 and path-2 are very similar to each other. After applying prestress, the difference between the stress values in the fiber and the interphase increase with the increase in the interphase’s elastic modulus. The axial stress in the RVE is different from the radial and circumferential stress values, because the prestress enlarges the stress differences between the fiber and the interphase.

The max and min stress values in the interphase are listed in Table 6. It can be observed that compared with the circumferential and axial stress values, the max radial stress value in the interphase is less sensitive to the interphase’s elastic modulus. With the increase in the elastic modulus of the interphase, the max radial stress values are changed much less. Without considering the prestress, the thermal residual circumferential and axial stress increases with a similar tendency. After considering the prestress, the circumferential stress in the interphase is inhibited. The axial stress value is changed from tensile stress to compressive stress by applying prestress.

### 4.6. Effects of Heterogeneous Elastic Modulus

**Figure 30:**
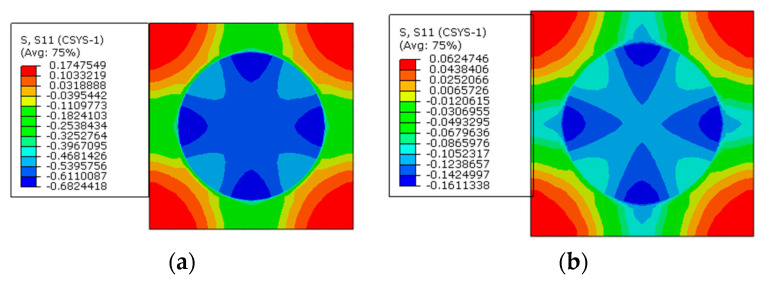
Radial stress distribution patterns of the model with heterogeneous elastic modulus (**a**) before applying prestress and (**b**) after applying prestress.

**Figure 31:**
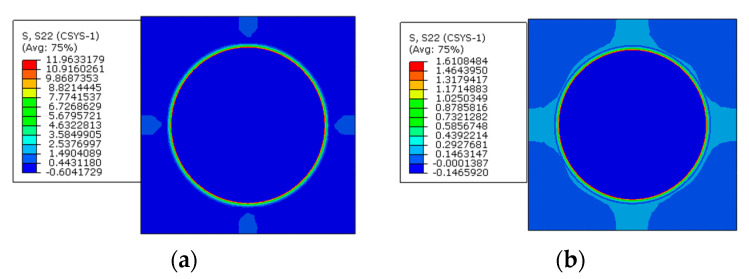
Circumferential stress distribution patterns of the model with heterogeneous elastic modulus (**a**) before applying prestress and (**b**) after applying prestress.

**Figure 32:**
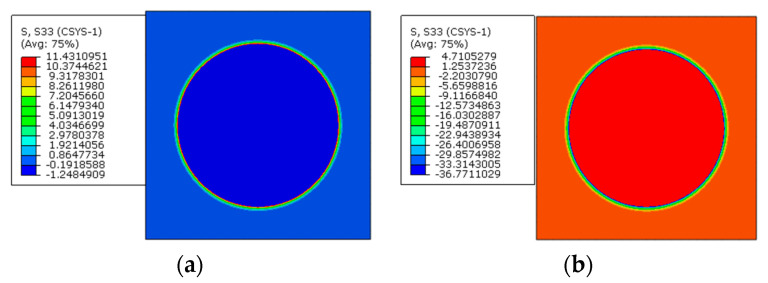
Axial stress distribution patterns of the model with heterogeneous elastic modulus (**a**) before applying prestress and (**b**) after applying prestress.

Models with four different elastic modulus distribution patterns of the interphase (Figure 2) are analyzed in this section. For the model with the first distribution case, it can be observed that the radial stress distribution patterns (Figure 30) are similar to the results of the model with the interphase elastic modulus equaling 50 GPa (Figure 24c,f). From the circumferential (Figure 31) and axial (Figure 32) stress distribution patterns, it can be observed that the heterogeneous elastic modulus contributes to the stress gradient in the interphase. The stress concentrates in the interphase near the fiber due to the large elastic modulus value.

**Figure 33:**
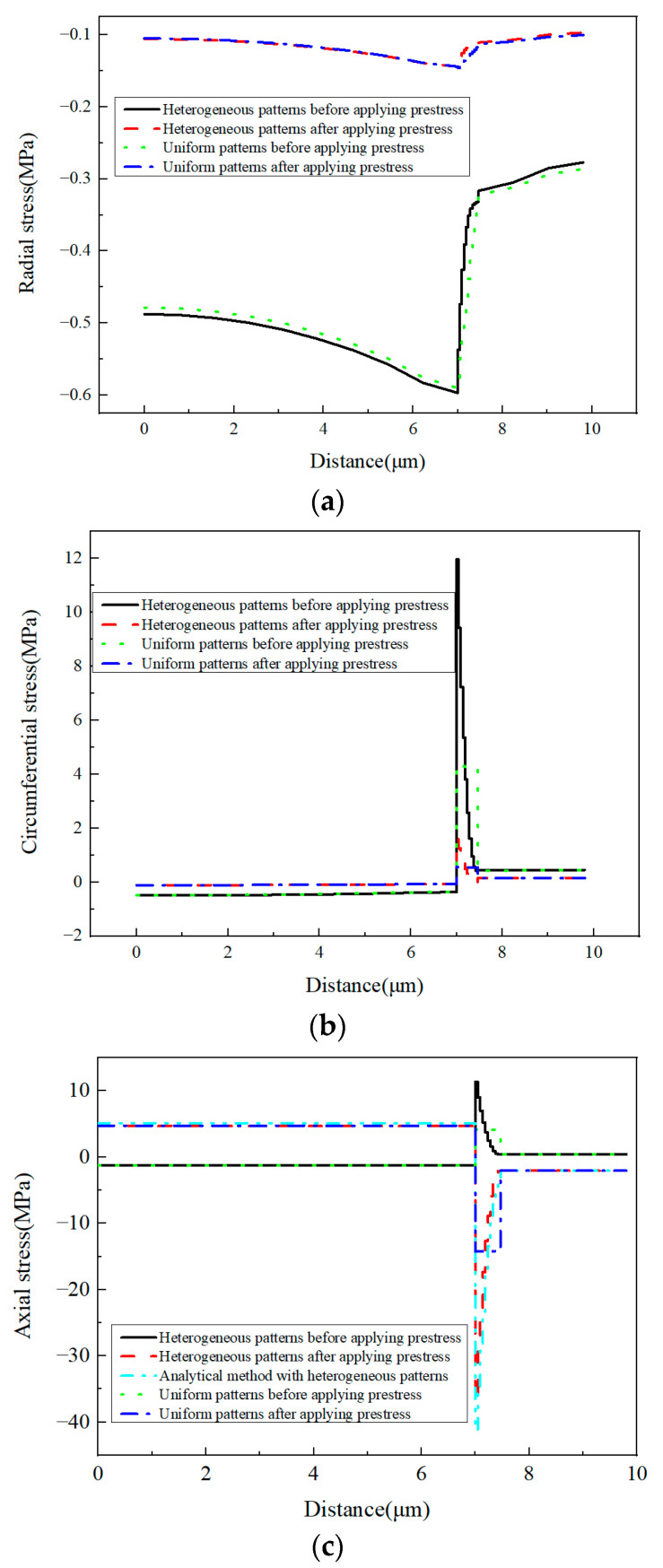
Stress values along path-1 with elastic modulus distribution case one. (**a**) Radial stress values; (**b**) circumferential stress values; (**c**) axial stress values.

The stress values in three different directions are shown in Figure 33 along path-1. The stress distribution patterns along the path are similar to the results shown in [34]. Comparing the stress values (Figure 33) along path-1 from the models with elastic modulus distribution case one with the corresponding homogenized elastic modulus value, it can be observed that the stress values in the fiber and matrix are almost the same, which means that the stress values of the fiber and the matrix can be predicted accurately from the model with equivalent homogenized elastic modulus.

However, due to the heterogenization of the elastic modulus, the radial stress gradient in the interphase is nonlinear. The effects of the heterogenous elastic modulus on the circumferential and axial stress values in the interphase are more serious than the effects on the radial stress values. The heterogeneous elastic modulus results in much larger circumferential and axial stress values in the interphase. The largest stress value induced by the heterogeneous elastic modulus is more than twice as large as the stress value of the model with the corresponding homogenized elastic modulus. Through averaging the heterogenous stress values in the interphase, the average stress values from the models with heterogenous elastic patterns are illustrated in Table 7. It can be observed that the average stress value is very close to the result of the corresponding model with the uniform elastic modulus. Furthermore, with the modified analytical method, the residual axial stress values in each interphase layer are also shown in Figure 33c. The similarity between the results from the analytical and numerical methods demonstrates the accuracy of the predicted stress values.

**Figure 34:**
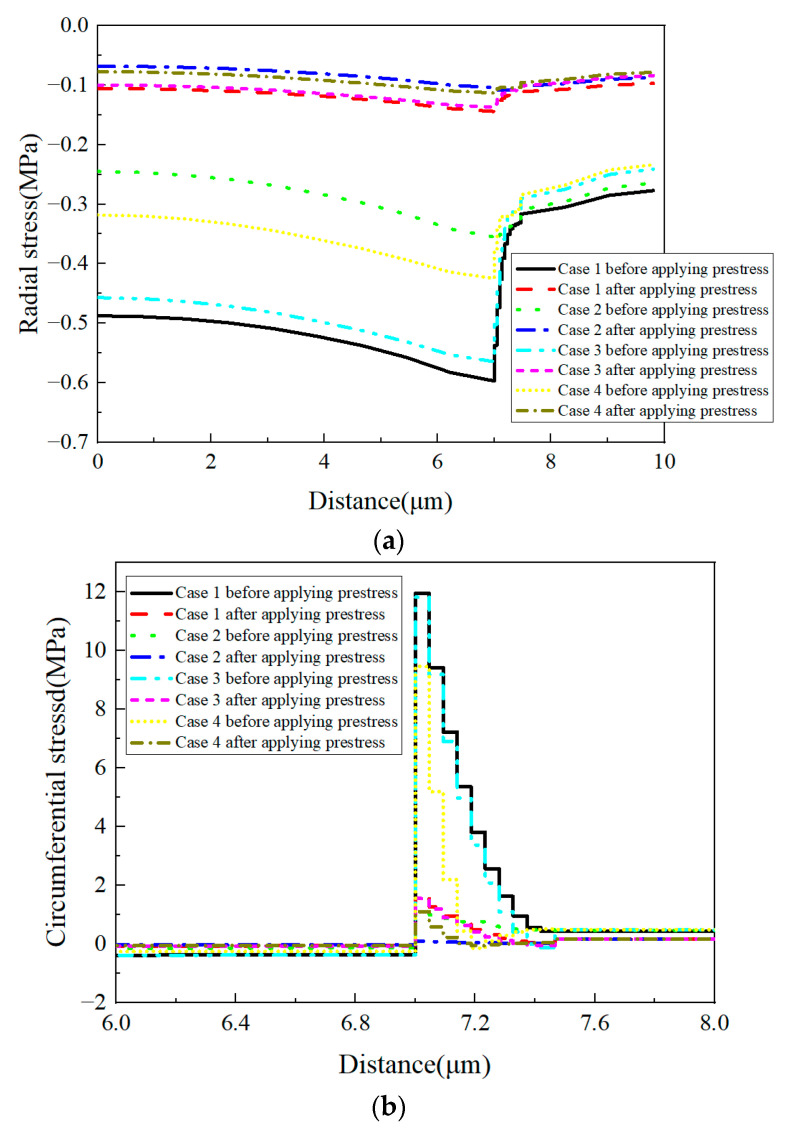

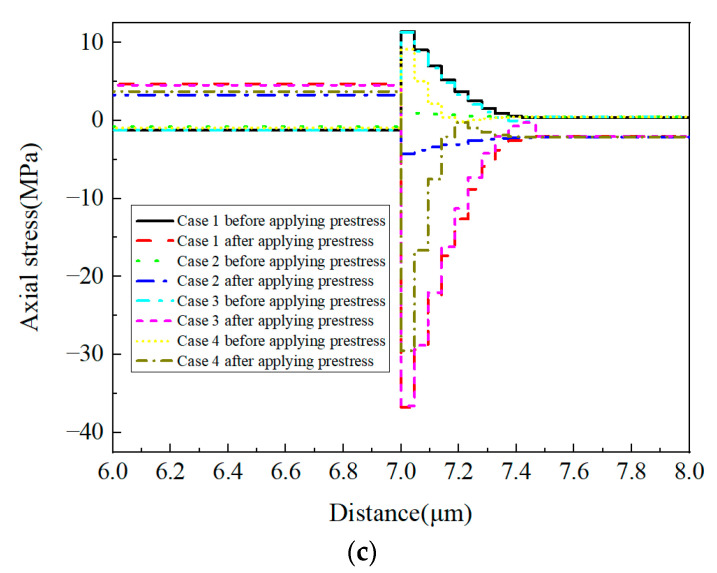
Stress values of the models with heterogeneous elastic modulus along path-1. (**a**) Radial stress values; (**b**) circumferential stress values; (**c**) axial stress values.

The stress values from the models with four different elastic modulus distribution patterns are shown in Figure 34 along path-1. From the radial stress distribution patterns (Figure 34a), it can also be observed that the stress values in the constituents are dependent on the equivalent interphase’s elastic modulus. The heterogenous elastic modulus has little influence on the radial stress distribution patterns. The application of prestress results in the decrease in the compressive stress in the fiber and interphase.

For the circumferential (Figure 34b) and axial (Figure 34c) stress values along path-1, the stress values in the fiber and matrix are also dependent on the equivalent elastic modulus of the interphase. With the heterogeneous interphase, the larger elastic modulus results in larger circumferential and axial stress. Thus, the distribution patterns of stress values in the interphase are almost consistent with the distribution patterns of the elastic modulus.

## 5. Conclusions

To obtain the residual stress distribution patterns of prestressed composites considering the interphase with FEM, the multi-step methodology is developed in this study. The thermal analysis is conducted first to obtain the thermal residual stress. Then, the equivalent thermal method for prestress application is modified and used to apply the prestress. Finally, the residual stress distribution patterns of prestressed composites are derived. The following conclusion can be drawn from the analyses.

1. Comparing the results predicted from the multi-step method with the results from the analytical method, it is demonstrated that the multi-step method can be used to predict the residual stress values of prestressed composites accurately.

2. Not only the axial stress, but also the radial and circumferential stress distribution patterns can be changed by the prestress level. The increase in the prestress level contributes to the increase in the difference between the axial stress values of the fiber and the matrix and the change of radial stress in the interphase from the compressive state to the tensile state. Thus, it is deduced that a large prestress level would result in the interphase debonding.

3. The increase in the CTE in the interphase also contributes to the change of stress distribution patterns along all the three directions, especially for the interphase. With the increase in the interphase’s CTE, the stress in the interphase tends to change from the compressive state to the tensile state. The change of the circumferential and axial stress is opposite, and the change rate of the circumferential and axial stress values is much larger than that of the radial stress.

4. The increase in the elastic modulus of the interphase has larger impacts on the fiber and the interphase than on the matrix. For the circumferential and axial stress values, due to the balance of the RVE, the increase in the tensile or compressive stress values in the interphase contributes to the increase in stress values in the fiber. For the radial stress, the increase in the elastic modulus results in the increase in compressive stress values in the fiber and the interphase.

5. The stress values in the matrix and the fiber predicted from the model with heterogeneous elastic modulus of the interphase are similar to the results from the model with corresponding uniform interphase elastic modulus. The heterogenous interphase elastic modulus contributes to the increase in circumferential and axial stress values in the interphase.

With the developed numerical methods, the effects of the residual stress on the progressive damage analysis of the prestressed composites could be evaluated with FEM under external mechanical loading conditions in the future.

## Figures and Tables

**Table 1:** Elastic parameters for the fiber and matrix [19].

Glass Fiber		Matrix	
Modulus, *E_f_* (GPa)	74	Modulus, *E_m_* (GPa)	3.35
Poisson’s ratio, *v_f_*	0.2	Poisson’s ratio, *v_m_*	0.35
Coefficient of Thermal Expansion, CTE*_f_* (/°C)	4.9 × 10^−6^	Coefficient of Thermal Expansion, CTE*_m_* (/°C)	58 × 10^−6^

**Table 2:** Axial stress values predicted with only prestress application considered.

Constituents	Analytical Method [27]	Multi-Step Method	Modified Equivalent Thermal Method
Fiber (MPa)	3.8	3.930	3.935
Matrix (MPa)	−2.5	−2.641	−2.645

**Table 3:** Axial stress values predicted with both polymer curing and prestress application considered.

Constituents	Analytical Method [11]	Numerical Method Developed in This Study
Fiber (MPa)	3.2	3.17
Matrix (MPa)	−2.14	−2.11

**Table 4:** Axial stress values predicted with interphase considered.

Constituents	Fiber (MPa)	Matrix (MPa)	Interphase (MPa)
Modified analytical method	3.03	−2.14	−1.22
Numerical method	3.06	−2.12	−1.24

**Table 5:** Max and min stress values in the interphase with different interphase CTE.

Interphase CTE	*α_i_* = 0.1*α_m_*		*α_i_* = *α_m_*		*α_i_* = 10*α_m_*	
Prestress Application	Before	After	Before	After	Before	After
Max radial stress	−0.06651	−0.01377	−0.05936	−0.01935	0.06374	0.2751
Max circumferential stress	−0.06842	−0.3614	0.3167	0.02148	4.194	3.988
Min circumferential stress	−0.2169	−0.4227	0.1841	−0.02173	3.931	3.684
Max axial stress	−0.07208	−1.607	0.3138	−1.221	4.202	2.764
Min axial stress	−0.2339	−1.672	0.1693	−1.268	4.173	2.638

**Table 6:** Max and min stress values in the interphase with different interphase elastic modulus.

	*E_i_* = 1.9 GPa		*E_i_* = 5 GPa		*E_i_* = 50 GPa	
MPa	Before	After	Before	After	Before	After
Max radial stress	−0.05936	−0.01935	−0.05274	−0.01739	−0.04885	−0.01848
Max Circumferential stress	0.3167	0.02148	0.919	0.08883	9.374	1.549
Min Circumferential stress	0.1841	−0.02173	0.7943	0.04928	8.693	1.409
Max axial stress	0.3138	−1.221	0.9051	−3.168	8.808	−29.06
Min axial stress	0.1693	−1.268	0.7602	−3.216	8.745	−29.09

**Table 7:** Average stress values in interphases for the models with heterogeneous elastic modulus.

Direction	Circumferential Stress		Axial Stress	
Prestress Application	Before	After	Before	After
Heterogenous elastic modulus (MPa)	4.391225	0.566071	4.233318	−14.2137
Uniform elastic modulus (MPa)	4.268806	0.556931	4.157911	−14.2131

## Data Availability

The data presented in this study are available on request from the corresponding author.
